# Significant response to Pembrolizumab for metastatic cutaneous squamous cell carcinoma in patient with Netherton syndrome

**DOI:** 10.1016/j.amsu.2022.104323

**Published:** 2022-08-03

**Authors:** Salem M. Tos, Bilal Nabeel Alqam, Narmeen Giacaman, Mohammad G. Ibdah, Mahmoud M.M. Gabajah, Abdallah Altell

**Affiliations:** a Al-Quds University, College of Medicine, Palestine; b Istishari Arab Hospital, Ramallah, State of Palestine, Palestine

## Abstract

**Background:**

Netherton syndrome is a rare autosomal recessive disease that presents with a triad of symptoms which include atopic diathesis, ichthyosis linearis circumflexa, and hair shaft abnormality termed “Bamboo Hair”. Netherton syndrome patients can develop cutaneous squamous cell carcinoma (cSCC) in unusually young age. Pembrolizumab is the first line treatment for locally advanced and recurrent/metastatic cSCC.

**Case presentation:**

A 44-year-old man with a history of Netherton syndrome and multiple skin squamous cell carcinoma was diagnosed with locally advanced and recurrent/metastatic cSCC two years ago. He was started on Pembrolizumab as a treatment for his cSCC. The immunotherapy course was well tolerated with no significant side effects including the expected immune related adverse events seen in patients treated with this medication. PET/CT scan showed significant regression of his disease consistent with partial response according to the response evaluation criteria in solid tumors.

**Discussion:**

Incurable and recurrent cSCC tends to metastasize, leading to an extremely poor long-term prognosis, and the treatment options for locally advanced or metastatic disease are few. Pembrolizumab, an immune checkpoint inhibitors (ICIs) showed a benefit in patients with various tumors including squamous cell carcinoma, but using this drug which is working by enhancing the immunity against tumor in patient with altered immunity like Netherton syndrome was a bit of a challenge, in terms of both effectiveness and safety.

**Conclusion:**

Pembrolizumab had a effective and safe treatment profile when it was used as a monotherapy for treating a Netherton syndrome patient diagnosed with locally advanced and recurrent/metastatic cSCC.

## Highlights

• Netherton syndrome presents with a triad of symptoms: atopic diathesis, ichthyosis linearis circumflexa, and hair shaft abnormality.
• Patients can also develop cutaneous squamous cell carcinoma (cSCC) in unusually young age.
• KEYTRUDA® (pembrolizumab) used to treat the cancer in this patient.

## 1 Introduction

Cutaneous squamous cell carcinoma (cSCC) is a cancer that arises due to malignant proliferation of epidermal keratinocytes [1]. It is considered the second most common non-melanoma skin cancer after basal cell carcinoma [2] and as for treatment it can be surgical or non-surgical with photodynamic therapy, topical with imiquimod or 5-fluorouracil, and laser ablation and immunotherapy [3]. Pembrolizumab, a PD-1 inhibitor, is a medication used in cancer immunotherapy that can be used as a first-line treatment for cSCC and has demonstrated an acceptable safety profile and clinically meaningful response when it was used as a monotherapy for locally advanced and recurrent/metastatic cSCC [4].

Netherton syndrome is a type of congenital ichthyosis, a rare autosomal recessive disease. It is caused by loss-of-function mutations in SPINK5 [5]. It is clinically characterized by a triad of symptoms that include atopic diathesis (elevated IgE), congenital ichthyosiform erythroderma (CIE) or ichthyosis linearis circumflexa (ILC), and hair shaft abnormality termed “Bamboo Hair” [6]. Patients with congenital ichthyosis including Netherton syndrome can develop cutaneous SCC in unusually young age [7].

There is absent data addressing treatment options for patients with Netherton syndrome and advances in cSCC. In the current paper, we present such a rare case treated with Pembrolizumab and report data with the outcome and tolerability for that patient. And Advanced CSCC patients are associated with a higher average cost than patients with resectable CSCC [8].

This case report has been reported in line with the SCARE Criteria [9].

## 2 Case presentation

A 44-year-old man, with a history of Netherton syndrome and multiple skin squamous cell carcinoma, came to outpatient clinic complaining of dry cough, dyspnea and squeezing chest pain of 3-month duration. The patient was born with scaly erythroderma, sparse hair growth except on the head and neck and seasonal allergy with elevated IgE level. He is a product of non-consanguineous healthy parents and family history revealed that he has a brother with a known case of Netherton syndrome.

**Fig. 1:**
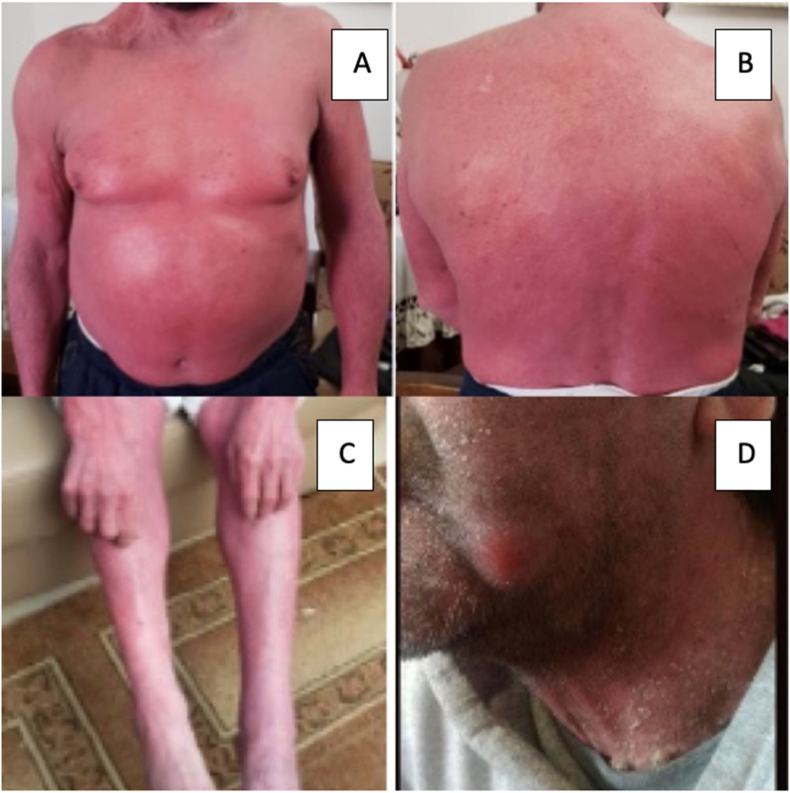
**A:** Ichthyosiform erythroderma (Generalized erythroderma and greasy, yellow-to-white scale on face and neck). **B, C, D:** Generalized erythroderma involving all body. (For interpretation of the references to colour in this figure legend, the reader is referred to the Web version of this article.)

The patient uses topical corticosteroid cream applied to his whole body daily after taking a shower to prevent the itching and reduce the redness since he was 13 years old (Fig. 1). Additionally, he takes oral antihistamine for his seasonal allergy.

The patient also has a long history of skin tumors. At the age of 36-years-old, the first mass appeared on his right lateral neck, but he ignored it until the age of 41-years-old as it became very ugly looking. He underwent surgical excision with flap rotation and free margins and histological evaluation of the skin mass revealed that it was cSCC. No further management or investigations were done. Since then and a year after, two local recurrent masses developed and were treated in the same manner.

Three months after the last local recurrence, the patient came to us complaining of dry cough that was severe enough to cause left direct inguinal hernia, dyspnea and squeezing chest pain. Meanwhile, during that year he had lost 10 kg without any changes in his diet.

**Fig. 2:**
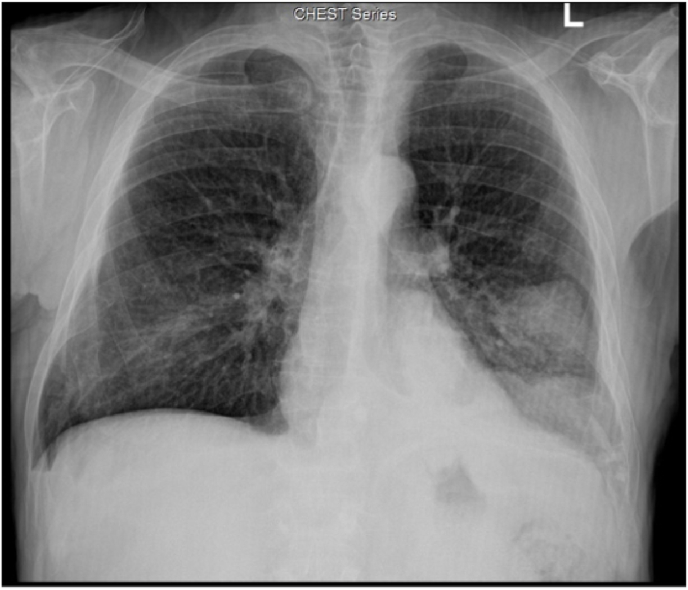
Chest-XR revealed well defined irregular rounded opacity with surrounding consolidation at the left lower lung zone.

Chest x-ray revealed well defined irregular rounded opacity measuring about 4.3 × 5 cm seen at the left lower lung zone, surrounded by a regional area of consolidation (Fig. 2).

**Fig. 3:**
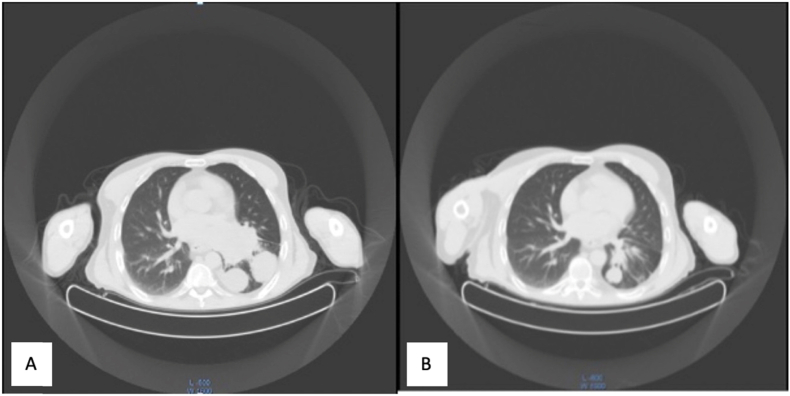
**A:** CT/PET scan at diagnosis: showed three left lung lesions. **B:** CT/PET scan 12 months of using keytruda: showed significant regression of these lesions.

Chest computed axial tomography (CT) scan performed showed three pulmonary mass lesions, the largest one measuring 8.7 × 8 × 6.5 cm in the left lower lobe extending to the anterior basilar segment and to the left infra-hilar region. The other two pulmonary lesions were in the left lower lobe within the medial and lateral base of segments, the larger of these measuring up to 4.2 cm in diameter. There was no evidence of any mass lesion in the right lung field nor pericardial or pleural effusion (Fig. 3A).

**Fig. 4:**
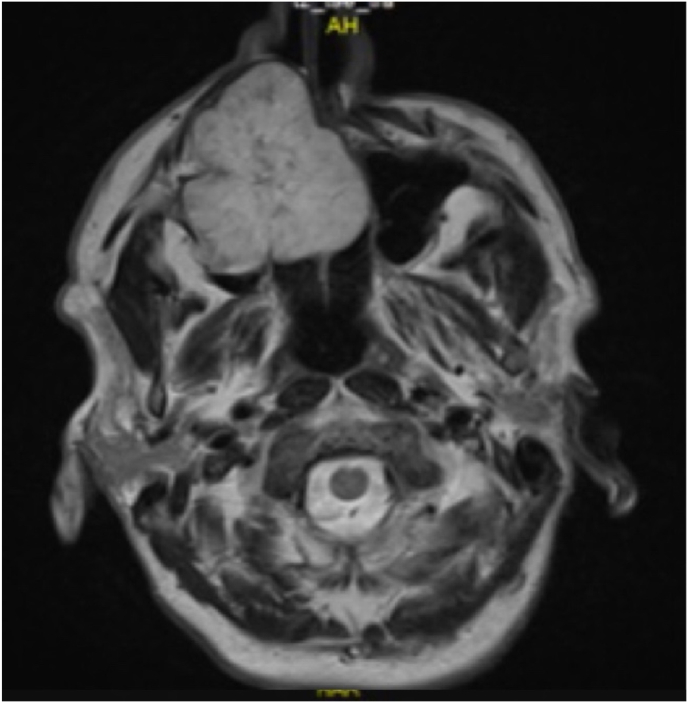
Brain MRI showed right maxillary sinus mass extending to the nasal cavity and two small osteolytic skull lesions.

Furthermore, a bronchoalveolar lavage and biopsy were performed revealing moderately differentiated SCC which were negative for both Thyroid transcription factor-1 (TTF-1) and Napsin A. A brain MRI showed right maxillary sinus mass extending to the nasal cavity and two small osteolytic skull lesions (Fig. 4).

He was started on Pembrolizumab as a treatment for his metastatic SCC. Till now, he had received 20 cycles of pembrolizumab (200 mg intravenously), fixed 3 weeks apart. During his treatment he hadn't develope any immune related adverse events and improving clinically after the third cycle. His cough and dyspnea were much better, and his appetite and weight improved during the treatment period.

In conclusion, the immunotherapy course was well tolerated with no significant side effects in the patient and the PET scan and CT scan done at 6 and 12 months after starting pembrolizumab showed significant regression of his disease consistent with partial response according to the Response Evaluation Criteria In Solid Tumors (RECIST v. 1.1). At the time of this report, pembrolizumab treatment is still ongoing, and he remains free of progression almost 15 months after starting pembrolizumab (Fig. 3B).

## 3 Discussion

Inherited ichthyoses are rare inherited disorders with skin manifestations caused by mutations in the genes involved in epidermal development. They include many diseases such as keratitis–ichthyosis–deafness (KID) syndrome, autosomal recessive congenital ichthyosis and Netherton syndrome. These patients seem to be prone to skin malignancies, including SCC, at an unusually young age. Hence, these patients are recommended to have routine skin cancer surveillance [10].

The exact mechanism of carcinogenesis in Netherton syndrome (NS) is unclear. It may be related to chronic infection, like HPV, chronic inflammation of the skin, or can be related to the use of UV light and immunosuppressive medication. A retrospective cohort study published in 2000 [11], aimed to study the presence of HPV infection in different skin lesions of three male NS patients and to investigate a possible association between HPV and malignancies in NS, the results showed that seven of 22 (31%) biopsies of the three NS patients were positive for HPV DNA, it also revealed that one patient who had five lesions which were diagnosed as cSCC, three of these five cSCC were positive for HPV. However, the patient's polymerase chain reaction (PCR) did not detect any HPV DNA in samples taken from the SCC masses.

Several findings of immunological abnormalities in NS, including dysfunction of memory B cells and natural killer cells, and mutations of SPINK5 that may affect T cell differentiation l eading to IgE overproduction [12]. The use of immunosuppressive medication in already immunosuppressed patients has been also associated with an increased incidence of skin cancers [13,14]. Topical steroid cream was used to reduce the redness and itchiness in the patient so it could be a possible cause for the masses that were found.

The patient who was a known case of Netherton syndrome wasn't following any routine skin cancer surveillance and was ignorant towards the growths on his skin that later appeared to be cSCC which had already metastasized to his lung, maxillary sinus, and skull bones. He was started on Pembrolizumab for his recurrent and metastatic cSCC.

Non-melanoma skin cancer (NMSC) constitutes one-third of all malignancies. NMSC includes basal cell carcinoma (80%) and cSCC (20%). Although cSCC represents about only 20% of NMSC, it has the ability to metastasize which is considered a threat and it is worth to mention that 20% of skin cancer deaths are attributable to cSCC [15]. Most patients with cSCC can be treated with surgical resection of the tumor.

Unfortunately, incurable and recurrent cSCC tends to metastasize leading to an extremely poor long-term prognosis of metastatic disease, and the treatment options for locally advanced or metastatic disease are few (they include platinum-based chemotherapy and off label use of cetuximab in addition to radiotherapy) [16]. The response usually lasts a short time, often accompanied by significant side effects, especially in elderly and frail subjects. For this condition, the 10-year survival rate is less than 20% for patients having regional lymph node metastasis and less than 10% for those patients having distant metastasis [17].

cSCC has a high tumor mutation burden (TMB) that results from UV radiation-induced DNA damage. Due to their high TMB, cSCC may be responsive to immunotherapy as treatment [4].

Pembrolizumab is an engineered humanized IgG4 mAb against PD-1 [18], and it is used as a new emerging tumor treatment known as an anti-cancer, being part of the family classified as immune checkpoint inhibitors (ICIs) [19]. It showed a benefit in patients with various tumors (e.g., advanced non-small-cell lung cancer), melanoma, urothelial carcinoma, squamous cell carcinoma of head and neck (SCCHN), and Merkel cell carcinoma [[20], [21], [22], [23], [24]].

Pembrolizumab showed clinically meaningful and durable antitumor activity with a manageable safety profile in patients with R/M cSCC based on the first interim analysis (IA). Thus, Pembrolizumab was approved by the USA FDA for R/M cSCC that is not curable by surgery or radiation [25].

In addition to its antitumor effect, it can potentially provoke immune-related adverse events (irAEs) across any organ as and these are major side effects of this drug, including skin, liver, gastrointestinal and endocrine inflammation [26,27]. A cohort study published in 2016 [26], involving 496 patient were screened for anti-PD-1 AEs. In total, 242 rare or unexpected side effects of nivolumab and pembrolizumab were documented in 138 patients (27.8%). Neurological, respiratory, musculoskeletal, cardiac, ocular and haematopoietic AEs occurred in 77 of the 138 patients. Some side effects are reported for the first time like cardiac arrhythmia, paresis, aphasia, a meningo-(radiculitis), and parkinsonoid syndrome. Furthermore, rare side effects like myasthenia gravis and polyradiculitis are also reported. however, in our patient, none of these events occurred after 16 months of using the anti-PD-1 drug even though he has immunological dysfunction as he is a Netherton syndrome patient.

To the best of our knowledge, this is the first case of using pembrolizumab for recurrent and metastatic cutaneous SCC in a patient with Netherton syndrome.

## 4 Conclusion

Pembrolizumab monotherapy, which enhances the immune response against tumor, demonstrated effective antitumor activity, clinically meaningful response, and acceptable safety parameters in primarily middle-aged man with metastatic cSCC, in spite the fact that the patient had an already immunity dysfunction since birth (Netherton syndrome in this case) due to uncertain mechanism, and also elevated IgE and eosinophils level due to seasonal allergy.

This is a first case of using Pembrolizumab for metastatic cSCC in patient with Netherton syndrome who uses topical steroid for his skin changes.

## Ethical approval

The study is exempt from ethical approval in our institution.

## Sources of funding

No funding or grant support.

## Author contributions

Study concept or design: Abdallah altell.

Writing the manuscript: Salem M. Tos, Bilal Nabeel Alqam, Narmeen Giacaman, Mohammad G. Ibdah and Mahmoud M. M. Gabajah.

Review & editing the manuscript: Salem M. Tos, Bilal Nabeel Alqam.

## Registration of research studies

Not applicable.

## Guarantor

Dr. Salem M. Tos.

## Consent

Written informed consent was obtained from the patient for publication of this case report and accompanying images. A copy of the written consent is available for review by the Editor-in-Chief of this journal on request.

## Provenance and peer review

Not commissioned, externally peer reviewed.

## Declaration of competing interest

The authors declare no conflicts of interest.
